# Analysis of Killer Cell Immunoglobulin-Like Receptor Genes and Their HLA Ligands in Inflammatory Bowel Diseases

**DOI:** 10.1155/2020/4873648

**Published:** 2020-09-19

**Authors:** Fereshteh Beigmohammadi, Mahdi Mahmoudi, Jafar Karami, Nooshin Ahmadzadeh, Nasser Ebrahimi-Daryani, Nima Rezaei

**Affiliations:** ^1^Rheumatology Research Center, Tehran University of Medical Sciences, Tehran, Iran; ^2^Rheumatology Expert Group (REG), Universal Scientific Education and Research Network (USERN), Tehran, Iran; ^3^Inflammation Research Center, Tehran University of Medical Sciences, Tehran, Iran; ^4^Department of Immunology, School of Medicine, Iran University of Medical Sciences, Tehran, Iran; ^5^Department of Gastroenterology and Hepatology, Tehran University of Medical Sciences, Tehran, Iran; ^6^Research Center for Immunodeficiencies, Children's Medical Center, Tehran University of Medical Sciences, Tehran, Iran; ^7^Department of Immunology, School of Medicine, Tehran University of Medical Sciences, Tehran, Iran; ^8^Network of Immunity in Infection, Malignancy and Autoimmunity (NIIMA), Universal Scientific Education and Research Network (USERN), Tehran, Iran

## Abstract

Genetic studies have illustrated that *killer cell immunoglobulin-like receptor* (*KIR*) genes could participate in various autoimmune disorders. We aimed to clarify the role of *KIR* genes, *HLA* ligands, HLA-KIR interactions, and their genotypes in inflammatory bowel disease (IBD) susceptibility. The study population was composed of 183 IBD subjects, comprising 100 ulcerative colitis (UC) patients, 83 Crohn's disease (CD) patients, and 274 healthy subjects. Polymerase chain reaction with sequence-specific primers (PCR-SSP) was used to evaluate the absence or presence of the 15 *KIR* genes, 5 *HLA* class I ligands, and 2 pseudogenes. We did not find any significant difference in allele frequency of *KIRs* and pseudogenes between IBD patients and healthy controls. In the case of *HLA* genes, there was a significant difference in *HLA-B-Bw4^Thr80^* frequency between UC patients and healthy controls (*P* = 0.03, OR = 0.06, 95%CI = 0.008-0.4). Furthermore, we found a significant difference in *HLA-C1^Asn80^* frequency between CD patients and healthy controls (*P* = 0.04, OR = 0.49, 95% CI = 0.3-0.8). In the full-array combination of *KIR* genes, there was no significant frequency difference between UC patients and healthy controls, while two KIR genotypes showed a significant susceptible association with CD. Our data do not support a strong role of NK cells in IBD susceptibility, but it does not rule out a role for KIR variability in IBD patients. However, there are some protective associations such as Bw4 alleles; these associations may be due to the interaction of the alleles to TCRs rather than KIRs.

## 1. Introduction

Inflammatory bowel diseases (IBDs) are categorized as chronic autoimmune diseases, which mostly target the gastrointestinal tract (GI). Ulcerative colitis (UC) and Crohn's disease (CD) are the two primary forms of IBDs [1–3]. The main parts involved in the UC are the rectum and colon, whereas the most part involved in CD is the ileum [4]. Although the etiology of IBDs is not completely understood, it has been shown that there are complex interactions between susceptible genes and environmental factors, which lead to defective inflammatory responses [5]. Environmental triggers such as microbiota in a genetically susceptible person may lead to the dysregulation of the immune system, and afterward, their interactions with immune and nonimmune cells can initiate the diseases [6].

The natural killer (NK) cells play important roles in the innate immune system and could be implicated in early responses against infected and transformed cells through cytokine production and direct toxicity [7, 8]. Signaling through inhibitory and activating receptors determines the activity and function of NK cells. The HLA class I molecules act as ligands for killer cell immunoglobulin-like receptors (KIRs), which are presented on the surface of NK cells [7]. KIRs are a group of transmembrane proteins and are mainly presented by NK cells and some groups of T cells. Interaction between KIRs and their corresponding HLA class I ligands could play a critical role in the NK cell function. Furthermore, it has been documented that these genetic combinations could be implicated in cancers and autoimmune and infectious diseases [9, 10]. The KIR receptors are encoded by a family of genes located on chromosome 19 which consists of 15 genes and two pseudogenes [11]. Depending on the intracellular length (long or short) and extracellular domains (2D or 3D), the *KIR* genes were categorized into 3 groups: activating KIR (aKIR) genes (*KIR2DS1*, *2DS2*, *2DS3*, *2DS4*, *2DS5*, and *3DS1*), inhibitory KIR (iKIR) genes (*KIR2DL1*, *2DL2*, *2DL3*, *2DL5a*, *2DL5b*, *3DL1*, *3DL2*, and *3DL3*), and pseudogenes (*2DP1* with no protein expression and *3DP1* with a putative protein product). However, KIR2DL4 has the ability to act as both an inhibitory and activating receptor [12]. Interaction of KIR receptors with their appropriate HLA ligands (*HLA-B* and *HLA-C* alleles) leads to either activating or inhibitory signals according to the presence of intracellular immunoregulatory tyrosine-based activating or inhibitory motifs [11].

Two types of KIR haplotypes exist: A and B. Haplotype A only has one activating KIR, *2DS4*, while haplotype B has various combinations of *2DS1*, *2DS2*, *2DS3*, *2DS4*, *2DS5*, and *3DS1*. *HLA-C* molecules are the main ligand for inhibitory KIRs. *HLA-C* has various variants; however, in the case of KIR, recognition could be reduced into two groups based on the amino acid that is located in the extracellular domain at position 80 [9, 10]. Variants belonging to group 1 of *HLA-C* alleles are those with asparagine at this position and presented as ligands for *KIR2DL2* and *2DL3*, while variants belonging to group 2 of *HLA-C* alleles are those with lysine at this position and presented as ligands for *KIR2DL1*. There is no known KIR for *HLA-B*-*Bw6*, but Bw4, another allelic form of *HLA-B*, is recognized by *KIR3DL1*. Inhibitory and activating KIRs are almost identical in the sequence of HLA-binding domains; however, there is a low affinity between activating KIRs and HLA-C. This issue proposes that these class I molecules could not be used as a common ligand for aKIRs. There is an important structural difference in the cytoplasmic region of inhibitory and activating KIRs; the iKIRs have a long immunoreceptor tyrosine-based inhibitory motif in their tails, whereas the potential aKIRs need to transfer their signaling via adapter molecules that possess ITAM structures. Therefore, the aKIRs need an adaptor protein to activate NK and NKT cells.

Studies documented that frequency of activating and inhibitory KIRs and their haplotypes is different in various people [10, 13–15]. The body of evidence proposed that *KIR* genes have an essential role in various diseases. Some combinations of KIR-HLA could modulate the immune response of NK and NKT [16, 17]. Genetic association studies have documented that there is a link between aKIRs and various autoimmune and inflammatory diseases [13–15]. Alteration in NK cell activity in the gut through KIR-HLA interactions may propose a regulatory mechanism for inflammation. Jones et al. showed that there are differences in KIR frequencies, presenting that KIR and their interactions with cognate HLA could be implicated in the UC susceptibility [13].

In our study, the frequency of 15 *KIR* genes, 5 *HLA* ligands, and 2 pseudogenes was evaluated in the UC, CD, and control groups. We aimed to clarify the role of *KIR* and *HLA* genes and their genotypes in IBD susceptibility. To our knowledge, no study has evaluated the frequency of *KIR* genes, HLA ligands, and also their interactions in the Iranian population with IBDs.

## 2. Materials and Methods

### 2.1. Study Population

The study population was made up of 183 IBD patients (100 UC patients (41 men and 59 women) and 83 CD patients (28 men and 55 women)) aged 44.3 ± 10.16 years (mean ± SD), who were attending the IBD outpatient clinic at Imam Khomeini Hospital, Tehran, Iran. The control group consisted of 274 unrelated randomly selected healthy controls (106 men and 168 women) aged 41.5 ± 12.11 years. All the subjects had Iranian racial background, and IBD patients were age-, sex-, and ethnicity-matched with the healthy control subjects. The diagnosis of UC [18] and CD [19] was according to the diagnostic guidelines and combination of histopathological, radiological, endoscopic, and biochemical tests. Controls had neither family history nor clinical manifestations of any type of autoimmune disease. Patients who had other types of colitis were ruled out of our study.

This study was performed based on the Declaration of Helsinki guidelines. Moreover, methods were carried out under the relevant regulations and guidelines by the mentioned institution. All the participants were informed of blood collection, DNA isolation, and genetic evaluation. In addition, the written informed consent was signed by all participants and/or their legal guardian/s. The ethics committee of the Tehran University of Medical Sciences approved this study.

### 2.2. DNA Extraction and Genotyping

Blood samples were gathered into tubes with ethylenediaminetetraacetic acid (EDTA). The standard phenol/chloroform method [20] was applied to extract genomic DNA from blood samples. The purity and quantity of DNA were measured with NanoDrop (Thermo Fisher Scientific, USA). For further investigation, concentration was optimized to 100 ng/*μ*L. In order to genotype DNA samples, PCR-SSP was used to evaluate the absence or presence of 15 *KIR* genes (*KIR2DS1*, *2DS2*, *2DS3*, *2DS4* (full-length allele, 2DS4 (full); variant alleles, 2DS4 (var)), *2DS5*, *3DS1*, *2DL1*, *2DL2*, *2DL3*, *2DL4*, *2DL5A*, *2DL5B*, *3DL1*, *3DL2*, and *3DL3* genes), 5 HLA class I ligands (*HLA-B-Bw4^Ile80^*, *HLA-B-Bw4^Thr80^*, *HLA-A-Bw4* (1 and 2), *HLA-C2^Lys80^*, and *HLA-C1^Asn80^*), and two pseudogenes (*2DP1* and *3DP1* (*3DP1-1* and *3DP1-2*)).

PCR conditions and primers were according to Tajik et al. [21, 22], Vilches et al. [23], Chainonthee et al. [24], Gagne et al. [25], our previous study [26], Bunce et al. [27], and Voorter et al. [28] (Supplementary file 1). G protein-coupled receptor 98 (*GPR98*), *HLA-DR*, and growth hormone genes 1 and 2 (*GH1* and *GH2*) were used as internal controls in PCR-SSP reactions (Supplementary file 1). Each PCR reaction had an internal control. The mixture employed to achieve a10 *μ*L volume reaction was consist of 1 *μ*L of PCR buffer (10x), 0.32 *μ*L (10 pmol) of internal control for each reverse and forward primer, 1.28 *μ*L (10 pmol) of reverse and forward specific primers for each KIR, 0.1 *μ*L (5 U/*μ*L) of Taq DNA polymerase, 0.25 *μ*L (10 mM) of deoxynucleoside triphosphate (dNTP), 0.32 *μ*L (50 mM) MgCl2, 5.13 *μ*L water, and 2 *μ*L of template DNA. PCR temperature cycling conditions were as follows: 2 min at 96°C (denaturation) and then 10 s at 94°C (10 cycles) and 60 s at 65°C, followed by 10 s at 94°C (denaturation, 20 cycles), 50 s at 61°C (annealing), 30 s at 72°C (final extension), and finally 10 min at 4°C. The PCR system ABI/2720 was used to amplify the PCR products (Applied Biosystems, Foster City, CA, USA). In order to amplify the *HLA-B-Bw4*, annealing temperature was increased to 65°C. After electrophoresis in 2% agarose gels containing ethidium bromide, PCR products were visualized under ultraviolet light.

### 2.3. Statistical Methods

Statistical analyses were carried out by SPSS version 22 (IBM Corp., Armonk, NY, USA). In order to compare the frequency of KIR genes and their HLA ligands between the case and control groups, Pearson's chi-squared test with continuity correction was applied. When the expected difference between the two groups was small, Fisher's exact test was applied. For estimations of strength of association, odds ratios (ORs) and 95% confidence intervals (CIs) were used. The Benjamini-Hochberg method was performed to control the false discovery rate (FDR) [29]. After correction for comparison, *P* values less than 0.05 (<0.05) were considered to be statistically significant. Pearson's chi-squared test with one degree of freedom was applied to confirm the Hardy–Weinberg equilibrium constant.

A geometric series was used in order to determine the specific genotype and its frequency. Genotype ID shows an exclusive number generated by the summation of members of a geometric series comprising products of each gene code (1 for positive and 0 for negative) multiplied by consecutive powers of 2: (first gene × 20) + (second gene × 21), +(third gene × 22), etc.

## 3. Results

### 3.1. Implication of KIR and HLA Genes in IBD Susceptibility

Primer sets (specific and internal) of the PCR-SSP assay for combined KIR-HLA genotyping are depicted in Supplementary file 1. Associations of *KIR* and *HLA* genes with IBDs (UC and CD) are depicted in Table 1. There were no significant differences in the frequency of inhibitory KIRs and pseudogenes between IBD patients and healthy subjects. Concerning aKIRs, the frequency of *KIR2DS4* (full) was significantly different between the UC and control groups (*P* = 0.02, OR = 0.53, 95% CI 0.3–0.9), and also, the frequency of *KIR2DS3* was significantly different between the CD and control groups (*P* = 0.048, OR = 1.64, 95% CI 1.0–2.7). These differences were no longer significant after the FDR correction. Indeed, there were no significant differences in the frequency of *KIR* genes between IBD patients and healthy controls. In the case of *HLA* genes, the frequency of *HLA-B-Bw4^Thr80^* was significantly different between the UC and control subjects, and this association was significant after FDR correction (*P* = 0.03, OR = 0.06, 95% CI 0.008–0.4). Furthermore, the frequency of *HLA-C1^Asn80^* and *HLA-B-Bw4^Thr80^* was significantly different between the CD and control groups, but after FDR correction, just the frequency of *HLA-C1^Asn80^* remained to be significant (*P* = 0.04, OR = 0.49, 95% CI 0.3–0.8) (Table 1).

### 3.2. Implication of KIR and/or HLA Genotypes on IBD Susceptibility

The association of KIR genotypes with IBDs is illustrated in Table 2. There was no significant difference between the UC and control groups in the full-array combination of the frequency of *KIR* genes. In the case of CD patients and healthy controls, KIR genotype no. 5 and 6 showed a significant susceptible association with CD (*P* = 0.01, OR = 8.7, 95% CI 1.7–45.8 and *P* = 0.001, OR = 33.2, 95% CI 4.1–266.3, respectively).

The association of *HLA* gene combinations in IBDs is illustrated in Table 3. We did not find any significant difference in the frequency of the gene combinations neither between the UC and control groups nor between CD and control groups. In the evaluation of aKIR and iKIR genotype frequencies, there were no significant differences between IBD patients and healthy controls (Tables 4 and 5).

### 3.3. Receptor-Ligand Interaction

Seventeen pair sets of KIR/HLA interaction in 2 possible conditions (absence and presence of interaction) were analyzed to clarify the role of these interactions in IBD susceptibility. Among all confirmed KIR/HLA pairs, there was no significant association between the UC and control groups.

There was a significant difference in the frequency of *KIR2DL3*^(+)^/*HLA.C1*^Asn(+)^ interaction between the CD patients and the control group (43 (52%) vs. 186 (67%), 0.51 (0.31-0.85), *P* = 0.009).

## 4. Discussion

Investigations on IBD patients have shown that the innate immune system plays critical roles against microbial infections through the initiation of an inflammatory response in the bowel [30]. One of these innate immune cells with an important role in GI is NK cell. NK cells are regulated through KIR-HLA interactions. Studies showed that these interactions were implicated in various autoimmune rheumatic diseases [31–33]. Nonetheless, results obtained about *KIR* genes and IBD susceptibility from different studies are controversial [34].

Determining the role of KIRs and HLA ligands in IBD susceptibility needs further investigations since NK cells have a vast repertoire of surface receptors, which are participating in NK cell activity [31].

During the past few years, many authors have been interested in the role of NK cells in IBDs [35–37]. Johansson et al. discussed the controversial roles of NK cells in the disease. In addition, they suggested that future investigations should be focused on the anatomical localization of NK cells and the cytokine environment [38]. Giacomelli et al. indicated that NK cells have a low level of killing activity when cocultured with PBMC of IBD patients. They proposed that this decreased activity could not be because of primary defects in NK cells since these NK cells were normal in killing activity. They suggested that inhibitory serum factors, likely from lymphocytes, could be produced in IBD patients, thereby leading to a low level of NK cell activity [39]. Another study has also shown that the NK cell cytotoxicity of UC patients is reduced [40].

There are reports that showed associations between the expression of surface receptors of NK cells and their cognate HLA ligands and autoimmune and infectious diseases [8]. For instance, ankylosing spondylitis is one of these rheumatic diseases in which the frequency of two *KIR* genes (*KIR2DL3* and *KIR2DL5*) and two HLA ligands (*HLA-B27* and *HLA-C2^Lys80^*) was significantly different between AS patients and healthy controls [41]. Moreover, *HLA-C^w4^* and *KIR2DS4* were associated with rheumatoid arthritis [42]. Our recent study did not show any significant differences in the frequency of *KIR* genes between BD patients and healthy controls. In the case of *HLA* genes, *HLA-B5*, *HLA-B51*, *HLA-B-Bw4^Ile80^*, and *HLA-C2^Lys80^* showed a susceptible association, while *HLA-C1^Asn80^* showed a protective effect against BD [26]. Jones et al. documented that *KIR2DS2* and *KIR2DL2* have a susceptible association with UC. In addition, they showed that the *KIR2DL3* in the presence of its ligand, *HLA-C1*, has a protective effect against UC [13]. Hollenbach et al. reported that the *KIR2DL2/3* has a susceptible association, while the C2 ligand has a protective effect on CD [43].

We evaluated the genetic diversity of KIRs and their corresponding ligands in the Iranian population with CD and UC. In the case of *KIR* genes, we reported that these genes are not associated with susceptibility to IBDs. Indeed, our results were in line with the studies of Saito et al. [44] and Wilson et al. [11], showing no significant association between *KIR* genes and CD patients. In the case of UC patients, our results were different from the mentioned studies. Indeed, each study tells a different story about the role of *KIR* genes in susceptibility to UC. The same data were obtained about the role of HLA class I with the disease susceptibility.

In our study, the *HLA-B-Bw4^Thr80^* showed a higher frequency in controls in comparison to UC patients and showed a protective role for the *HLA-B-Bw4^Thr80^* in UC patients. Furthermore, the *HLA-C1^Asn80^* also showed a higher frequency in controls in comparison to CD patients and showed a protective role for the *HLA-C1^Asn80^* in CD patients (Table 1). Therefore, we can only assume that there is a potential relationship between *HLA* genes and IBD diseases. However, none of these HLA genotype combinations were significant between the control and patient groups (Table 3). In the case of aKIR and iKIR genotypes, we did not show any significant association between these genotypes and IBD susceptibility (Tables 4 and 5). Gene-gene interaction analysis showed that the interaction between *KIR2DL3* and *HLA.C1^Asn^* has a susceptible role in CD.

## 5. Conclusions

Collectively, our data do not support a strong role of NK cells in IBD susceptibility, but it does not rule out a role for KIR variability in IBD patients. However, there are some protective associations such as Bw4 alleles (present in 1% of UC patients and 4% of CD patients versus 14% of controls). These associations may be due to the interaction of these alleles to TCRs rather than KIRs.

## Figures and Tables

**Table 1:** Comparison between *KIR* and *HLA* gene frequencies in the IBD and control groups.

*KIR* alleles	UC, *N* (%)	CD, *N* (%)	Control, *N* (%)	UC vs. control	Adj. *P*^a^	Odds ratio (95% CI)	CD vs. control	Adj. *P*^a^	Odds ratio (95% CI)
				*P* value			*P* value		
Inhibitory									
2DL1	99 (99)	81 (98)	267 (97)	0.37	0.78	2.59 (0.3-21.3)	0.94	1	1.06 (0.2-5.2)
2DL2	64 (64)	55 (66)	167 (61)	0.59	0.98	1.14 (0.7-1.8)	0.38	1	1.25 (0.7-2.1)
2DL3	85 (85)	74 (83)	245 (89)	0.24	0.78	0.67 (0.3-1.3)	0.94	1	0.97 (0.4-2.1)
2DL4	100 (100)	83 (100)	274 (100)	—	—	—	—	—	—
2DL5	69 (69)	61 (73)	215 (78)	0.059	0.59	0.61 (0.4-1.1)	0.34	1	0.76 (0.4-1.3)
2DL5A	100 (100)	83 (100)	135 (49)	—	—	—	—	—	—
2DL5B	55 (55)	53 (64)	167 (61)	0.30	0.78	0.78 (0.5-1.2)	0.63	1	1.13 (0.7-1.9)
3DL1	90 (90)	74 (89)	254 (93)	0.39	0.78	0.71 (0.3-1.6)	0.29	1	0.64 (0.3-1.5)
3DL2	100 (100)	83 (100)	274 (100)	—	—	—	—	—	—
3DL3	100 (100)	83 (100)	274 (100)	—	—	—	—	—	—
Activating									
2DS1	70 (70)	61 (73)	172 (63)	0.19	0.24	1.38 (0.8-2.2)	0.07	0.24	1.64 (0.9-2.8)
2DS2	67 (67)	55 (66)	173 (63)	0.49	0.49	1.18 (0.7-1.9)	0.60	0.63	1.14 (0.7-1.9)
2DS3	48 (48)	41 (49)	102 (37)	0.06	0.16	1.55 (0.9-2.5)	0.048	0.24	1.64 (1.0-2.7)
2DS4 (full)	19 (19)	21 (25)	84 (31)	0.02	0.14	0.53 (0.3-0.9)	0.35	0.63	0.76 (0.4-1.3)
2DS4 (var)	85 (85)	62 (75)	217 (79)	0.21	0.24	1.48 (0.8-2.7)	0.38	0.63	0.77 (0.4-1.4)
2DS5	47 (47)	33 (40)	101 (37)	0.07	0.16	1.51 (0.9-2.4)	0.63	0.63	1.13 (0.7-1.9)
3DS1	53 (53)	41 (49)	124 (45)	0.18	0.24	1.36 (0.8-2.1)	0.51	0.63	1.18 (0.7-1.9)
Pseudogene									
2DP1	99 (99)	81 (98)	265 (97)	0.25	0.48	3.36 (0.4-26.8)	0.68	0.68	1.4 (0.3-6.5)
3DP1-1	24 (24)	18 (22)	80 (29)	0.32	0.48	0.76 (0.5-1.3)	0.18	0.68	0.67 (0.4-1.2)
3DP1-2	94 (100)	82 (99)	259 (95)	—	—	—	0.13	0.27	4.69 (0.6-36.5)
HLA alleles									
HLA-C1^Asn80^	76 (76)	50 (60)	207 (76)	0.93	1	1.02 (0.6-1.7)	0.007	0.04	0.49 (0.3-0.8)
HLA-C2^Lys80^	66 (66)	57 (69)	200 (73)	0.18	0.36	0.71 (0.4-1.2)	0.44	0.52	0.81 (0.5-1.4)
HLA-B-Bw4^Thr80^	1 (1)	3 (4)	38 (14)	0.006	0.03	0.06 (0.008-0.4)	0.017	0.051	0.23 (0.07-0.77)
HLA-B-Bw4^Ile80^	53 (53)	51 (61)	149 (54)	0.81	1	0.94 (0.6-1.5)	0.25	0.37	1.34 (0.8-2.2)
HLA-A-Bw4-1	21 (21)	25 (30)	85 (31)	0.058	0.17	0.59 (0.3-1.0)	0.10	0.2	0.6 (0.4-1.1)
HLA-A-Bw4-2	96 (96)	76 (92)	274 (100)	—	—	—	—	—	—

^a^FDR-adjusted *P* value for multiple testing using the Benjamini-Hochberg method. A *P* value less than 0.05 was statistically significant. HLA: human leukocyte antigen; KIR: killer cell immunoglobulin-like receptor; IBD: inflammatory bowel disease; UC: ulcerative colitis; CD: celiac disease.

**Table 2:** KIR genotypes in normal individuals and IBD patients.

KIR genotype	KIR genes																				Patient (%)	Control (%)	*P* value	OR (95% CI)
	Inhibitory KIR										Activating KIR							Pseudogene						
	2DL1	2DL2	2DL3	2DL4	2DL5	2DL5A	2DL5B	3DL1	3DL2	3DL3	2DS1	2DS2	2DS3	2DS4 F	2DS4 V	2DS5	3DS1	2DP1	3DP1-1	3DP1-2				
Ulcerative colitis vs. control																								
1	+	+	+	+	+	+	+	+	+	+	+	+	−	−	+	+	+	+	+	+	3 (3%)	8 (3%)	0.97	1.0 (0.2-3.9)
2	+	−	+	+	+	+	−	+	+	+	+	−	−	−	+	+	+	+	−	+	8 (8%)	23 (9.2%)	0.95	0.4 (0.4-2.2)
3	+	+	+	+	+	+	+	−	+	+	+	+	+		−	+	+	+	−	+	2 (2%)	7 (2.6%)	0.76	0.8 (0.2-3.8)
Crohn's disease vs. control																								
1	+	+	+	+	+	+	+	+	+	+	+	+	−	−	+	+	+	+	+	+	3 (3.6%)	8 (3%)	0.75	1.2 (0.3-4.8)
2	+	+	+	+	+	+	+	+	+	+	+	+	+	−	+	+	+	+	−	+	2 (2.4%)	14 (5.4%)	0.31	0.46 (0.1-2.1)
3	+	−	+	+	+	+	−	+	+	+	+	−	−	−	+	+	+	+	−	+	5 (6%)	23 (9.2%)	0.48	0.69 (0.2-1.9)
4	+	+	+	+	+	+	+	−	+	+	+	+	+	−	−	+	+	+	−	+	4 (4.8%)	7 (2.6%)	0.30	1.9 (0.5-6.7)
5	+	−	+	+	+	+	−	+	+	+	+	−	+	−	+	−	+	+	−	+	5 (6%)	2 (0.7%)	0.01	8.7 (1.7-45.8)
6	+	+	+	+	+	+	+	+	+	+	+	+	+	−	+	−	−	+	−	+	9 (10.8%)	1 (0.4%)	0.001	33.2 (4.1-266.3)

A *P* value less than 0.05 was statistically significant. Genotype frequency less than 2% is not included. KIR: killer cell immunoglobulin-like receptor; CD: Crohn's disease; UC: ulcerative colitis.

**Table 3:** Human leukocyte antigen (HLA) genotypes in normal individuals and IBD patients.

HLA genotype	HLA gene/allele					Patients (%)	Control (%)	*P* value	OR (95% CI)
	HLA-C1^Asn80^	HLA-C2^Lys80^	HLA-B-Bw4^Thr^	HLA-B-Bw4^Ile80^	HLA-A-Bw4				
Ulcerative colitis vs. control									
1	+	+	−	+	+	3 (3%)	22 (8%)	0.09	0.35 (0.1-1.2)
2	+	+	−	+	−	23 (23%)	40 (14.6%)	0.056	1.74 (0.9-3.1)
3	+	−	−	+	−	10 (10%)	31 (11.3%)	0.72	0.87 (0.4-1.8)
4	+	+	−	−	−	14 (14%)	32 (11.7%)	0.55	1.23 (0.6-2.4)
5	−	+	−	−	−	10 (10%)	24 (8.8%)	0.71	1.16 (0.5-2.5)
Crohn's disease vs. control									
1	+	+	−	+	+	4 (4.8%)	22 (8%)	0.33	0.58 (0.2-1.7)
2	+	+	−	−	+	5 (6%)	19 (6.9%)	0.77	0.86 (0.3-2.4)
3	−	+	−	−	+	5 (6%)	4 (1.5%)	0.03	4.33 (1.1-16.5)
4	+	+	−	+	−	15 (18.1%)	40 (14.6%)	0.44	1.29 (0.7-2.5)
5	+	−	−	+	−	10 (12%)	31 (11.3%)	0.85	1.07 (0.5-2.3)
6	+	+	−	−	−	5 (6%)	32 (11.7%)	0.14	0.48 (0.2-1.3)
7	−	+	−	−	−	11 (13.3%)	24 (8.8%)	0.23	1.59 (0.7-3.4)

A *P* value less than 0.05 was statistically significant. Genotype frequency less than 2% is not included.

**Table 4:** Activating KIR (aKIR) genotypes in normal individuals and IBD patients.

HLA genotype	HLA gene/allele							Patients (%)	Control (%)	*P* value	OR (95% CI)
	2DS1	2DS2	2DS3	2DS4 F	2DS4 V	2DS5	3DS1				
Ulcerative colitis vs. control											
1	+	+	+	−	+	+	+	13 (13%)	17 (6.6%)	0.13	1.74 (0.8-3.6)
2	−	+	−	+	+	−	−	3 (3%)	19 (7.4%)	0.16	0.41 (0.1-1.4)
3	−	−	−	+	+	−	−	3 (3%)	15 (5.8%)	0.33	0.53 (0.1-1.9)
4	−	−	−	−	+	−	−	10 (10%)	33 (13.7%)	0.58	0.81 (0.4-1.7)
Crohn's disease vs. control											
1	+	+	−	−	+	+	+	6 (7.2%)	12 (4.6%)	0.30	1.70 (0.6-4.7)
2	+	−	−	−	+	+	+	6 (7.2%)	23 (9.2)	0.73	0.85 (0.3-2.1)
3	+	+	+	−	+	−	−	13 (15.6%)	26 (10.5%)	0.11	1.77 (0.8-3.6)
4	−	−	−	−	+	−	−	4 (4.8%)	33 (13.7%)	0.06	0.37 (0.1-1.1)

A *P* value less than 0.05 was statistically significant. Genotype frequency less than 2% is not included.

**Table 5:** Inhibitory KIR (iKIR) genotypes in normal individuals and IBD patients.

HLA genotype	HLA gene/allele										Patients (%)	Control (%)	*P* value	OR (95% CI)
	2DL1	2DL2	2DL3	2DL4	2DL5	2DL5A	2DL5B	3DL1	3DL2	3DL3				
Ulcerative colitis vs. control														
1	+	+	+	+	+	+	+	+	+	+	28 (28%)	59 (22%)	0.19	1.41 (0.8-2.4)
2	+	+	−	+	+	+	+	+	+	+	8 (8%)	11 (4%)	0.12	2.08 (0.8-5.3)
3	+	−	+	+	+	+	−	+	+	+	12 (12%)	44 (16%)	0.33	0.71 (0.4-1.4)
Crohn's disease vs. control														
1	+	+	+	+	+	+	+	+	+	+	26 (31%)	59 (22%)	0.07	1.82 (1.1-3.1)
2	+	+	−	+	+	+	+	+	+	+	7 (8%)	11 (4%)	0.12	2.17 (0.8-5.8)
3	+	−	+	+	+	+	−	+	+	+	12 (14%)	44 (16%)	0.69	0.87 (0.4-1.7)
4	+	+	+	+	+	+	+	−	+	+	7 (8%)	10 (3.6%)	0.08	2.40 (0.8-6.5)

A *P* value less than 0.05 was statistically significant. Genotype frequency less than 2% is not included.

## Data Availability

All data generated or analyzed during this study are available upon request.

## Supplementary Materials

Supplementary Materials Supplementary file 1: the sequence of primer sets (specific and internal) of the PCR-SSP assay for combined KIR-HLA genotyping.
