# Supplementary material for: Analysis of Killer Cell Immunoglobulin-Like Receptor Genes and Their HLA Ligands in Inflammatory Bowel Diseases
Source: J Immunol Res. 2020 Sep 19;2020:4873648. doi: 10.1155/2020/4873648 (PMC7520679; doi:10.1155/2020/4873648)
Supplement: Supplementary Materials — Supplementary file 1: the sequence of primer sets (specific and internal) of the PCR-SSP assay for combined KIR-HLA genotyping. [file 4873648.f1.pdf]

Journal name: Journal of Immunology Research

Title: Analysis of killer-cell immunoglobulin-like receptors genes and their HLA ligands in inflammatory bowel diseases

Supplementary file 1. Primer sets and internal controls for combined KIR-HLA genotyping by PCR–SSP assay

**Primer Sequence for Gene amplification**					
**Reaction**	**Gene**	**Forward primer (5'–3')**	**Reverse primer (5'–3')**	**Size (bp)**	**References**
**1**	*2DL1*	TTGGTCAGATGTCATGTTTGAA	TCCCTGCCAGGTCTTGCG	143	(23)
**2**	*2DL2*	AAACCTTCTCTCTCAGCCCA	GCCCTGCAGAGAACCTACA	142	(23)
**3**	*2DL3*	ACAAGACCCTCAGGAGGTGA	GCAGGAGACAACTTTGGATCA	160	(23)
**4**	*2DL4*	TCAGGACAAGCCCTTCTGC	GACAGGGACCCCATCTTTC	130	(23)
**5**	*2DL5A*	GCGTACGTCACCCTCCCG	ACTTCTAGGCCCATCACTCC	314	(21)
**6**	*2DL5B*	CGTCACCCTCCCATGATGTA	ACTTCTAGGCCCATCACTCC	308	(21)
**7**	*2DS1*	GTAGGCTCCCTGCAGGGA	ACAAGCAGTGGGTCACTTGAC	148	(25)
**8**	*2DS2*	CTGCACAGAGAGGGGAAGTA	CAGAGGGTCACTGGGAGC	177	(21)
**9**	*2DS3*	ACCTTGTCCTGCAGCTCCT	AGCATCTGTAGGTTCCTCCT	160	(23)
**10**	*2DS4 (full)*	CAGCTCCCGGAGCTCCTA	TGACGGAAACAAGCAGTGGA	224	(21)
**11**	*2DS4 (var)*	CTTGTCCTGCAGCTCCATC	TGACGGAAACAAGCAGTGGA	202	(21)
**12**	*2DS5*	TGATGGGGTCTCCAAGGG	TCCAGAGGGTCACTGGGC	125	(25)
**13**	*3DL1*	TGAGCACTTCTTTCTGCACAA	TAGGTCCCTGCAAGGGCAA	129	(21)
**14**	*3DL2*	AAACCCTTCCTGTCTGCCC	TGGAAGATGGGAACGTGGC	134	(21)
**15**	*3DL3*	GCAATGTTGGTCAGATGTCAG	AGCCGACAACTCATAGGGTA	199	(23)
**16**	*3DS1*	TCCATCGGTTCCATGATGCG	GACCACGATGTCCAGGGGA	111	(23)
**17**	*2DP1*	ACATGTGATTCTTCGGTGTCAT	GTGAACCCCGACATCTGTAC	167	(21)
**18**	*3DP1 (full)*	GGTGTGGTAGGAGCCTTAG	GAAAACGGTGTTTCGGAATAC	280	(23)
**19**	*3DP1 (var)*	CGTCACCCTCCCATGATGTA	GAAAACGGTGTTTCGGAATAC	395	(23)
**20**	*HLA-C1^{Asn80}*	GAGGTGCCCGCCCGGCGA	CGCGCAGGTTCCGCAGGC	332	(22)
**21**	*HLA-C2^{Lys80}*	GAGGTGCCCGCCCGGCGA	CGCGCAGTTTCCGCAGGT	332	(22)
**22**	*HLA-B-Bw4^{Thr80}*	GGAGCGAGGGGACCGCAG	GTAGTAGCGGAGCGCGGTG	344	(22)
**23**	*HLA-B-Bw4 ^{Ile80}*	GAGCGAGGGGACCGCAG	GTAGTAGCGGAGCGCGATC	343	(22)
**24**	*HLA-A-Bw4*	TGGCGCCCCGAACCCTCG	GCTCTGGTTGTAGTAGCGGA	456	(22)
		AACCCTCCTCCTGCTACTCTT	GCTCTGGTTGTAGTAGCGGA	446	
**25**	*HLA-B5*	ACCGAGAACCTGCGGAT	CGTTCAGGGATGTAATCT	401	(27)
**26**	*HLA-B51*	GGAGTATTGGGACGAAC	CGTTCAGGGATGTAATCT	451	(27)
**27**	*HLA-B27*	GCTACGTGGACGACACG	GTCTGTGCCTTGGCCTT	142	(28)
		GGTCTCAACCCTCCAGA	GGAGCCACTCCACGCAC	234	
**Primer sequence for internal controls**					
**Genes**		**Forward primer (5'–3')(Gene)**	**Reverse primer (5'–3')(Gene)**	**Size (bp)**	**References**
**all KIRs except *KIR2DL5A/ B* and *KIR2DS5***		GCCTTCCCAACCATTCCCTTA (*GH1*)	TCACGGATTTCTGTTGTGTTTC (*GH1*)	429	(21)
KIR2DL5A, 2DL5B, 3DP1, HLA-A-Bw4, HLA-C1^{Asn80}, HLA-C2^{Lys80}, HLA-B-Bw4^{Ile80}		GCCTTCCCAACCATTCCCTTA (*GH2*)	GTCCATGTCCTTCCTGAAGCA (*GH2*)	1070	(21)
KIR2DS5		GAGGTAACTGTGCTCACGAACAGC (*HLA-DR*)	GGTCCATACCCCAGTGCTTGAGAAG (*HLA-DR*)	283	(23)
HLA-B-Bw4^{Thr80}		ATGGATCAGCCCAGCTGTCA (*GPR98*)	TCCTGCATTATGGCCCATTG (*GPR98*)	141	(24)

HLA; Human Leukocyte antigen, SSP-PCR; Single Specific Primer-Polymerase Chain Reaction, KIR; Killer-cell immunoglobulin-like receptor
